# Cedratvirus getuliensis replication cycle: an in-depth morphological analysis

**DOI:** 10.1038/s41598-018-22398-3

**Published:** 2018-03-05

**Authors:** Ludmila Karen dos Santos Silva, Ana Cláudia dos Santos Pereira Andrade, Fábio Pio Dornas, Rodrigo Araújo Lima Rodrigues, Thalita Arantes, Erna Geessien Kroon, Cláudio Antônio Bonjardim, Jônatas Santos Abrahão

**Affiliations:** 1 Departamento de Microbiologia, Instituto de Ciências Biológicas, Universidade Federal de Minas Gerais, Belo Horizonte, Minas Gerais Brazil; 2 Centro de Microscopia da Universidade Federal de Minas Gerais, Belo Horizonte, Minas Gerais Brazil

## Abstract

The giant viruses are the largest and most complex viruses in the virosphere. In the last decade, new members have constantly been added to this group. Here, we provide an in-depth descriptive analysis of the replication cycle of Cedratvirus getuliensis, one of the largest viruses known to date. We tracked the virion entry, the early steps of virus factory and particles morphogenesis, and during this phase, we observed a complex and unique sequential organization of immature particle elements, including horseshoe and rectangular compartments, revealed by transverse and longitudinal sections, respectively, until the formation of the final ovoid-shaped striped virion. The genome and virion proteins are incorporated through a longitudinal opening in the immature virion, followed by the incorporation of the second cork and thickening of the capsid well. Moreover, many cell modifications occur during viral infection, including intense membrane trafficking important to viral morphogenesis and release, as evidenced by treatment using brefeldin A. Finally, we observed that Cedratvirus getuliensis particles are released after cellular lysis, although we obtained microscopic evidence that some particles are released by exocytosis. The present study provides new information on the unexplored steps in the life cycle of cedratviruses.

## Introduction

The study of giant viruses has been intensified after the isolation of Acanthamoeba polyphaga mimivirus, a virus of outstanding dimensions, capable of infecting amoebas of the genus *Acanthamoeba*^1^. Since then, the intense prospection and improvement of isolation techniques has made possible the discovery of new viruses^2,3^. The presence of these viruses has been observed in rather diverse environments, such as water, soil, sewage, and clinical samples, as well as in extreme environments, including permafrost and soda lakes, for example^4–6^. These discoveries have revealed a wide diversity and variety of species not previously observed in the virosphere, challenging the concepts and paradigms concerning the canonical definition of viruses^7^. Currently, the International Committee of Taxonomy of Viruses (ICTV) officially recognizes two families of giant virus of amoebas: *Mimiviridae* and *Marseilleviridae*. In addition to these families, other giant viruses (not assigned yet) have been isolated, such as Faustovirus and Kaumoebavirus, the first giant viruses described to replicate in *Vermoamoeba vermiformes*^8,9^. The tupanviruses, recently isolated from Brazilian environments, present a complex virion structure, with a mimivirus-like capsid attached to a long tail, and these viruses replicate in a broad range of protists (unpublished data). Other isolated viruses, such as Pandoravirus, Pithovirus, Mollivirus and Cedratvirus, also have atypical virion morphologies, exhibiting amphora-shaped, spherical or ovoid structures^4,6,10,11^.

Among these viruses, the cedratvirus has an ovoid viral particle, morphologically similar to that of pithovirus but presenting two corks, one at each apex^4,10^. The first Cedratvirus, A11, was isolated from environmental samples from Algeria^10^. Then, a second isolate, Cedratvirus lausannensis, was recovered from a water treatment plant in Morsang-sur-Seine, France^12^. Through an extensive prospective study, we isolated the first cedratvirus from Brazil, named Cedratvirus getuliensis. Although studies on the prospection of giant viruses have advanced over the years, enabling the isolation of new viruses, information regarding their biology remains scarce. In the present study, we present an in-depth investigation of the replication cycle of Cedratvirus getuliensis (C. getuliensis). Through transmission electron microscopy and biological assays using different pharmacological inhibitors, we elucidated different steps of the replication cycle. We provided the first evidence of a complex and unique sequential organization of immature particles elements, including transverse-sectioned horseshoe and longitudinal-sectioned rectangular compartments, until the formation of the final striped, ovoid-shaped virion. Moreover, many cell modifications occur during viral infection, raising questions about the role of some organelles during the replication of Cedratvirus getuliensis. Amorphous particles were observed in many cells, similar to those previously observed for Pithovirus, but these particles were homogeneously diffused throughout the host cytoplasm, suggesting that deformed particles are naturally formed by Cedratvirus getuliensis. Finally, we observed that Cedratvirus getuliensis particles are released after cellular lysis, although we obtained microscopy evidence that some particles are released by exocytosis. These results provide new information on the unexplored steps in the life cycle of cedratviruses.

## Material and Methods

### **V**irus isolation, cell culture, production and titration

Cedratvirus getuliensis was previously isolated in 2017 from sewage samples collected in the city of Itaúna, Minas Gerais, Brazil. After isolation, the virus genome was sequenced, and subsequent bioinformatics analyses were developed; we observed high homology and synteny among the genomes of Cedratvirus getuliensis and other Cedratviruses (in preparation). For co-culture and isolation procedures, *Acanthamoeba castellanii* cells (ATCC 30010) were cultivated in Peptone-yeast extract with glucose (PYG)^13^ medium supplemented with 25 mg/ml amphotericin B (Fungizone; Cristalia, São Paulo, Brazil), 500 U/ml penicillin (Schering-Plough, Brazil) and 50 mg/ml gentamicin (Schering-Plough, Brazil). A total of 7 × 10E6 cells was infected with *C.* *getuliensis* at a multiplicity of infection (MOI) of 0.01 and incubated at 32 °C. After the appearance of a cytopathic effect, the cells and supernatants were collected, with sterile serological pipettes, stored in conic sterile tubes and the viruses were subsequently purified through ultracentrifugation with a 40% sucrose cushion at 36,000 *g* for 1 h. After purification, the viruses were serially diluted, and multiple replicate samples of each dilution were inoculated into *A. castellanii* monolayers. After 72–96 h of incubation, the amoebas were analyzed to determine whether infection occurred. Based on these data, the virus titers were determined using the endpoint method^13,14^.

### Entry and traffic membrane assays

In these experiments, we first evaluated the primary mechanism used by C. getuliensis to enter *A. castellanii* cells. For that we used different chemical inhibitors in order to investigate different endocytic pathways commonly explored by viral particles to enter in host cells, such as cytochalasin D – a phagocytosis inhibitor, chloroquine – clathrin and caveolin -dependent of acidification pathways inhibitors, and 5-(N-ethyl-N-isopropyl) amiloride (EIPA) – a specific macropinocytosis inhibitor. Cytochalasin D and chloroquine had already been confirmed as inhibitors of endocytic pathways in *Acanthamoeba*. However, the micropinocytosis inhibition effect induced by EIPA (observed in other systems) remains to be molecularly investigated in *Acanthamoeba*. A total of 5 × 10^5^ *A. castellanii* cells was pre-treated with 2 μM of cytochalasin (Sigma-Aldrich, United States), 100 μM of chloroquine (Sigma-Aldrich, United States) or 1 μM of EIPA (Sigma-Aldrich, United States). The cytotoxicity of the inhibitors was tested in *Acanthamoeba* and the choice by inhibitors concentrations was based on previous studies^15–22^. After 1 h, the cells were infected with C. getuliensis at an MOI of 5. Control groups of untreated infected amoebas were also prepared. Thirty minutes post-infection, cells and supernatant were collected and centrifuged at 800 g per 10 minutes. The resultant pellet was washed three times with Page’s amoeba saline (PAS)^13^. After, cells were submitted to three rounds of freezing and thawing, to allow the viral particles release, and then subjected to titration using the endpoint method^13,14^. In parallel, the supernatant of cytochalasin assay was also submitted to titration for comparison.

To evaluate the role of cell membranes in the viral replication cycle, 5 × 10^5^ *A. castellanii* cells were also infected with C. getuliensis at an MOI of 5. Thirty minutes post-infection, the amoebas were washed with PAS and then transferred to 6-well microplates containing 1 mL of PYG medium and maintained at 32 °C. After 1 h, brefeldin A (BFA), an inhibitor of membrane traffic, was added at a final concentration of 10 μM, and at 8 and 24 h post-infection, the amoebas were collected for TEM analysis and titration, respectively.

All experiments were performed in triplicate. Graphs were constructed using GraphPad Prism version 7.00 for Windows (GraphPad Software).

### Transmission electron microscopy and Scanning electron microscopy

For transmission electron microscopy (TEM), 7 × 10^6^ *Acanthamoeba castellanii* cells were subjected to an asynchronous viral infection using a low MOI of 0.1, and 24 hours post-infection they were recovered and pelleted for 10 min at 800 g. The pellet was washed twice with 0.1 M phosphate buffer (pH 7.4) and fixed with 2.5% glutaraldehyde in 0.1 M phosphate buffer for 1 h at room temperature. The pellet was then washed twice with 0.1 M phosphate buffer and resuspended in the same buffer. After repelleting, the amoebas were embedded in Epon resin by using a standard method, as follows: 2 h of fixation in 2% osmium tetroxide, five washes in distilled water, overnight incubation in uranyl acetate 2% at 2–8 °C, two washes in distilled water, 10 min dehydration in increasing ethanol concentrations (35%, 50%, 70%, 85%, 95% and 100% ethanol), 20 min incubation in acetone and embedding in EPON resin. Ultrathin sections were subsequently analyzed under transmission electron microscopy (TEM; Spirit Biotwin FEI-120 kV).

For scanning electron microscopy assays, 10 µL of purified particles of C. getuliensis were added to round glass coverslips covered with poly-l-lysine and fixed with 2.5% glutaraldehyde in 0.1 M cacodylate buffer for at least 1 h at room temperature. The same procedure was performed to observe *Acanthamoeba* cell interactions with C. getuliensis during the early (1 h.p.i) and late stages (24 h.p.i.) of infection. The samples were washed three times with 0.1 M cacodylate buffer and post-fixed with 1.0% osmium tetroxide for 1 h at room temperature. After a second fixation, the samples were washed three times with 0.1 M cacodylate buffer and immersed in 0.1% tannic acid for 20 min. The samples were then washed in cacodylate buffer and 10 min dehydrated by serial passages in ethanol solutions (35%, 50%, 70%, 85%, 95% and 100%). Samples were subsequently subjected to critical point drying using CO_2_, placed in stubs and metalized with a 5 nm gold layer. The analyses were completed using scanning electron microscopy (FEG Quanta 200 FEI).

## Results

### Cytochalasin impacts the incorporation of cedratvirus getuliensis particles by *Acanthamoeba castellanii* cells

**Figure 1:**
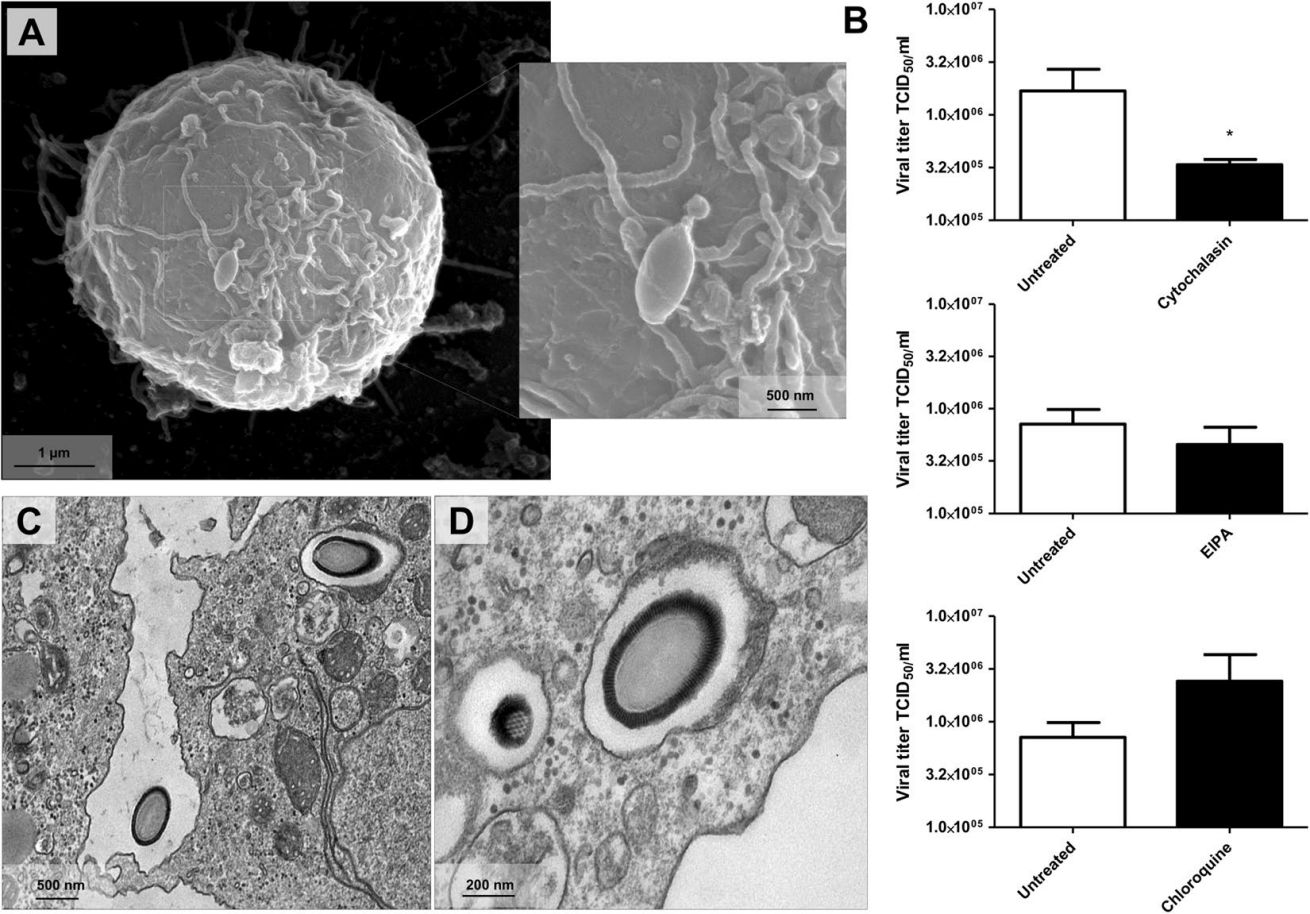
Cedratvirus getuliensis entry in Acanthamoeba castellanii cells. (**A**) Scanning microscopy showing a C. getuliensis particle attached to an *Acanthamoeba* cell. (**B**) The impact of different inhibitors of endocytic pathways in C. getuliensis entry. Treatment of amoebas with cytochalasin D reduced Cedratvirus getuliensis virion incorporation, indicating that particles can enter amoebas by phagocytosis. (**C**) and (**D**) TEM of C. getuliensis particles inside vesicles that strong resemble phagosomes.

Upon discovery of the first cedratviruses^10^, analyses involving transmission electron microscopy and scanning electron microscopy^12^ revealed the presence of particles with a similar morphology presented by other described viruses, such as pandoravirus and pithovirus. In addition to the morphological similarity, other aspects involving the replication cycle of these viruses were extrapolated and applied to characterize the cedratviruses, such as the internalization of viral particles in amoeba cells by phagocytosis. Our data indicate that Cedratvirus getuliensis can explore phagocytic pathways to enter *A. castellanii* cells, since the titration of pellet cells pretreated with cytochalasin D revealed a significantly decrease (p-value = 0.0385) in the viral titer, when compared to the untreated cells (Fig. 1A and B). Corroborating with those results, when we performed the titration of the supernatant, we observed a higher viral titer for samples pretreated with cytochalasin D, evidencing that a significant number of particles were not phagocytosed (p-value = 0.0243). Transmission electron microscopies of infected particles also corroborate this hypothesis, once C. getuliensis particles could be observed inside vesicles that strong resemble phagosomes (>500 nm), which is consistent with previous studies in which phagocytosis was investigated in amoebas (Fig. 1C and D)^23,24^. In contrast to cytochalasin D, pretreatment with EIPA did not result in a significant reduction in viral titer, indicating that the macropinocytosis is not essential for Cedratvirus getuliensis entry (Fig. 1B). However, as the effects of EIPA has not been previously studied in *Acanthamoeba*, the entry of cedratvirus getuliensis by macropinocytosis cannot be ruled out. In addition, some works have demonstrated that cytochalasin can also interfere on macropinocytosis, that’s why a in depth characterization of EIPA in *Acanthamoeba* would be important. Interestingly, we also observed a strong biological tendency of viral title increasing when *Acanthamoeba* cells were treated with chloroquine, an inhibitor of clathrin and caveolin pathways (Fig. 1B).

### Cedratvirus getuliensis infection induces the formation of an electron-lucent viral factory and causes cytoplasmic modifications involving different organelles

**Figure 2:**
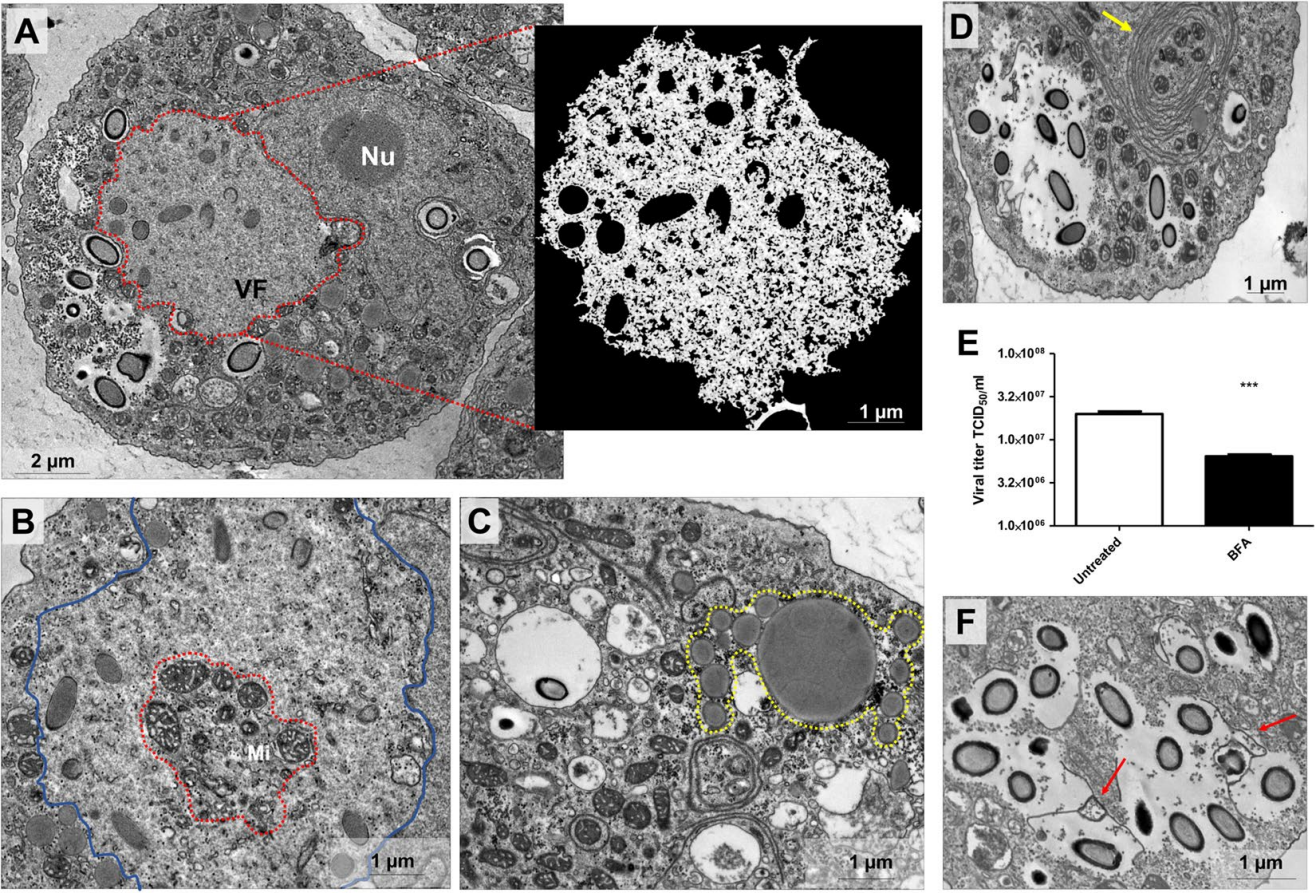
Electron-lucent viral factory and cytoplasmic modifications induced by Cedratvirus getuliensis modification. (**A**) C. getuliensis presents an electron-lucent viral factory (contoured in red and in detail) not easily distinguished from the rest of the cytoplasm and observed at the perinuclear region. Different stages of viral particle morphogenesis could also be observed within the viral factory. (**B**) Abundant presence of mitochondria inside (contoured in red) the viral factory (contoured in blue). (**C**) Lysosomal accumulation and polarization in the host cytoplasm (contoured in yellow). (**D**) Intensified membrane traffic in the host cytoplasm (yellow arrow). (**E**) Treatment with BFA reduces the viral titer after 24 hours of infection. (**F**) Infected cells treated with BFA presented membrane degradation after 8 hours of infection. VF: Viral factory. Nu: Nucleus. Mi: Mitochondria. Image A-right was obtained by TEM and graphically highlighted by using IOS image visualization software.

The replication of many viruses occurs in subcellular microenvironments designated viral factories that originate from the reorganization of cytoskeleton, organelles and cellular membrane compartments^25^. Similarly, the morphogenesis of cedratviruses, as other giant viruses^15,26^, occurs in a viral factory located in the cytoplasm of host cells. Using TEM images of Cedratvirus getuliensis replication cycle, we observed the presence of an evident viral factory that in general is as large as the cellular nucleus. Different from mimiviruses, which present an electron-dense viral factory divided into different parts (one related to genome replication and morphogenesis and another one associated with fibrils acquisition) and are easily distinguished from the rest of the host cytoplasm, the C. getuliensis viral factory is electron-lucent and does not exhibit well-defined zones, thus preventing its prompt distinction from the remaining cytoplasm (Fig. 2A)^26-28^. Moreover, the morphogenesis of C. getuliensis progeny could be observed in the periphery and in the middle of the viral factory, where some electron-dense structures were observed, in contrast to the results observed for mimiviruses, for which the final assembly of new particles occurs at the edge of the factory (Fig. 2A).

Interestingly, we observed that the C. getuliensis viral factory is typically situated at the perinuclear region. During the cycle, the nucleus was consistently present and apparently not affected by the virus, different from that described for pandoraviruses, in which some nuclear disorganization with numerous membrane invaginations were observed in infected cells^11,29^. In addition, during C. getuliensis replication, some absorbing cellular alterations were observed (Fig. 2B,C and D). One alteration was the abundant presence of mitochondria inside and around the viral factory (Fig. 2B). Another interesting change was the intense accumulation and polarization of structures that resemble lysosomal vesicles in the host cytoplasm, particularly during the late steps of the cycle (Fig. 2C). Finally, we also observed exacerbated membrane traffic (Fig. 2D), revealed as important for the morphogenesis and/or exocytosis release of virions, upon the treatment of amoebas with BFA, which significantly impacts the viral titer after 24 hours of infection (Fig. 2E). TEM images also showed a decrease of membrane traffic, as well as membrane degradation in BFA-treated cells, after 8 h of infection (Fig. 2F).

### Cedratvirus getuliensis morphogenesis involves the complex and unique sequential organization of immature particles

**Figure 3:**
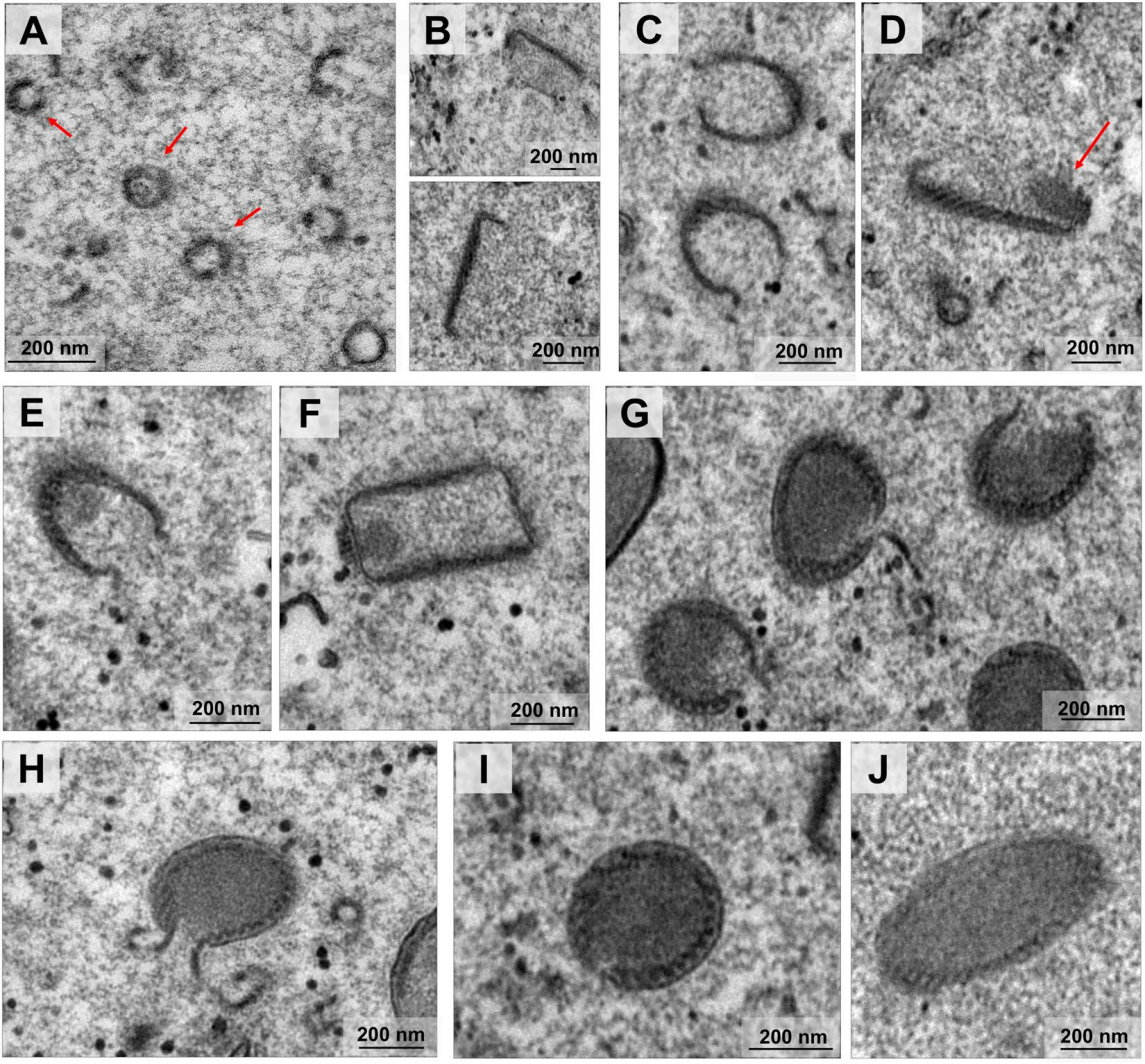
Cedratvirus getuliensis morphogenesis involves the occurrence of subsequent complex structures. (**A**) First discernible viral structures showing a crescent-shaped capsid precursor. (**B**) Longitudinal sections revealed the longitudinal-elongation of the particle and capsids assuming a staple-shaped conformation. (**C**) Transversal sections showed empty capsids with a horseshoe conformation. (**D**) Longitudinal sections showed staple-shaped with the first cork evident (red arrow). The particle may be an open cylinder at this moment, since transversal-sectioned particles appears as rectangles (**F**) and transversal-sectioned particles still reveals horseshoe-like structures (**E**). Progressive filling of the capsid (**G,H**). (**J**) Complete closure of the capsids.

C. getuliensis morphogenesis is a complex process involving the formation of subsequent structures that could be clearly visualized as electron-dense materials within and at the periphery of the viral factory in TEM images (Fig. 3). TEM images should be analyzed with cautious, since 2D perspective can lead to misinterpretation. However, the obtained images suggest that the first discernible viral particle structures are crescent-shaped ~100 nm precursors developed in the middle of viral factory (Fig. 3A). Similar structures, described as open membrane intermediates or precursors, have been also observed during Vaccinia virus, Mimivirus and African Swine fever virus replication, suggesting the occurrence of a common assembly steps for NCLDVs^30–33^. The following observed differentiation is the longitudinal elongation of the particle (~600 nm), when the precursor capsid assumes a staple-shaped conformation, as visualized by longitudinal sections (Fig. 3B). Transversal sections revealed empty capsids with a similar horseshoe conformation and an evident striated wall, a characteristic feature of pithoviruses as also observed^4,34^ (Fig. 3C). At this stage, only one cork region is clearly visible in the longitudinal cut, at the pole where the morphogenesis probably started (Fig. 3D). The particle appears to be an open cylinder at this moment, since longitudinal-sectioned particles appear as rectangles (Fig. 3F) and transversal-sectioned particles still reveal horseshoe-like structures (Fig. 3E). Next, we observed a progressive filling of the capsid (Fig. 3G,H and I), followed by the complete closure of the capsid (Fig. 3J) and the emergence/incorporation of the second cork.

**Figure 4:**
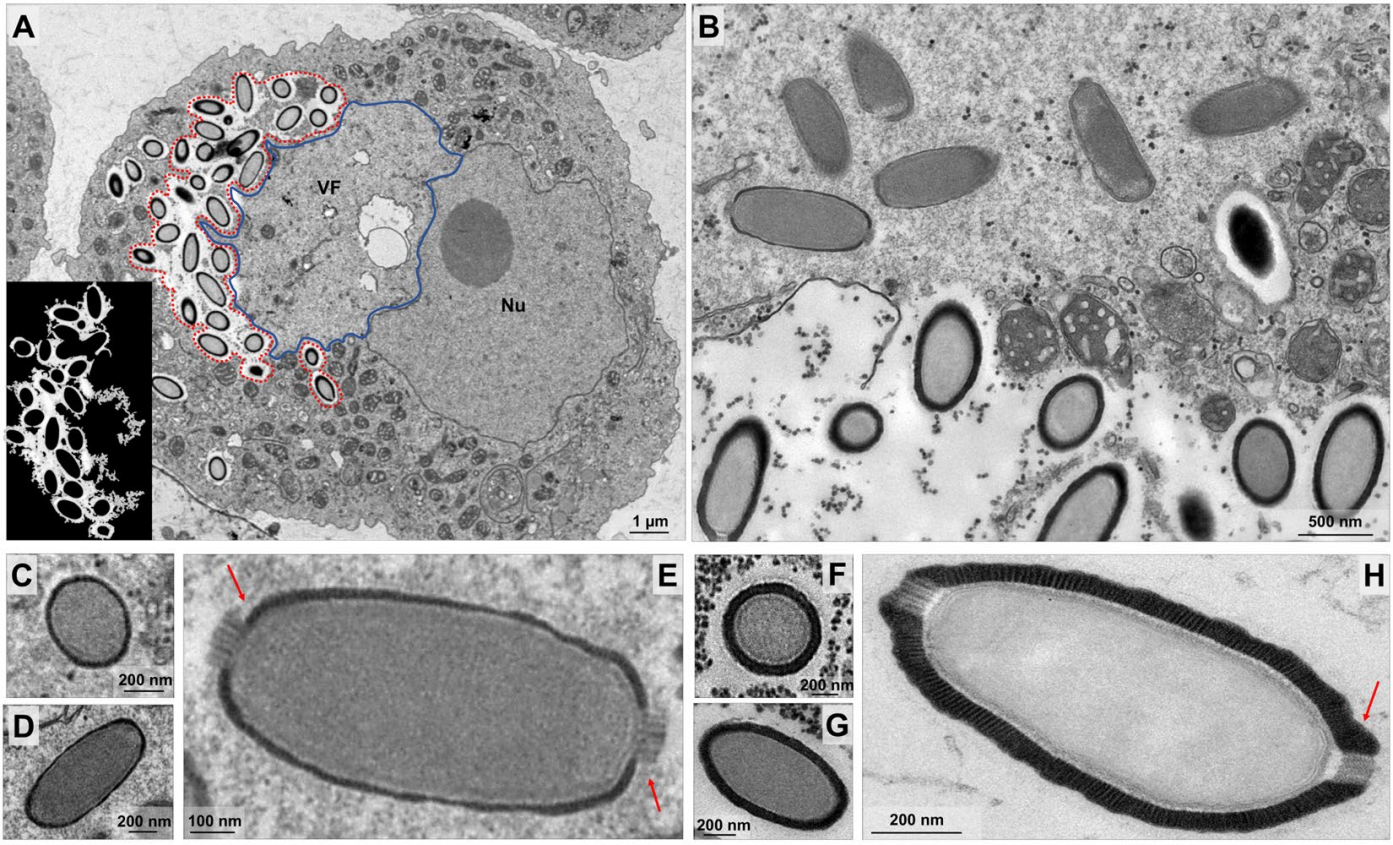
Particle wall thickening after capsids closure. (**A**) Viral particles suffer differentiation related to the thickness in a specific area at the edge of the viral factory (contoured in red and in detail). (**B**) Viral factory periphery evidencing the capsid wall thickness. Cross (**C**) and longitudinal (**D**) sections of capsids presenting a thin thickness and the corks not completely laterally covered (**E**) (red arrows). (**F**) and (**G**) The capsids become thicker with the progression of maturation and acquire the same thickness presented by both corks (**H**) (red arrow). VF: Viral factory. Nu: Nucleus. Image A-left was obtained by TEM and graphically highlighted by using IOS image-visualize software.

Following the total closure of the capsid, we observed that this structure undergoes some degree of differentiation related to the capsid wall thickness. Immediately after capsid closure, some ovoid particles are observed in the periphery of the viral factory and particle thickening occurs in an area at the edge of or surrounding the viral factory (Fig. 4A and B). Initially, the capsid presents a thin wall and the two corks are not completely laterally covered (Fig. 4C,D and E). As the maturation progresses, the capsid wall becomes thicker until it acquires the same thickness presented by both corks (Fig. 4F,G and H).

### Misshapen Cedratvirus getuliensis particles could be observed during virus morphogenesis

**Figure 5:**
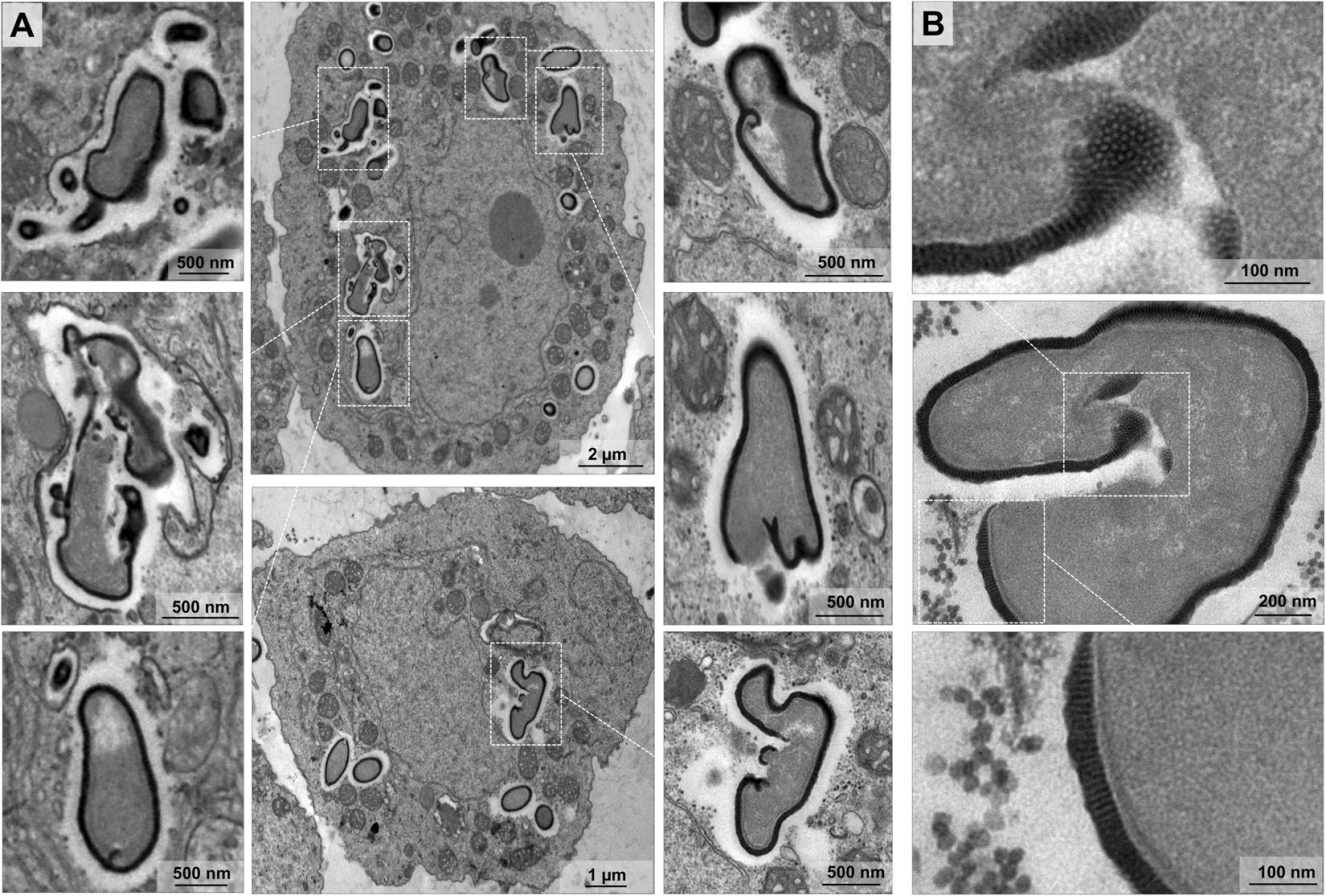
Misshapen structures observed during C. getuliensis multiplication. (**A**) and (**B**) Amorphous structures resembling defective particles and composed by portions of corks, striated capsids, membrane and electron-dense material could be visualized in different regions of the host cytoplasm alongside mature virus.

We also observed the appearance of some misshapen structures as blobs comprising portions of corks, capsids, membrane and electron-dense material (Fig. 5A and B). These unusual structures have previously been described by Legendre and colleagues in the Pithovirus sibericum replication cycle as “possible reservoirs of partially organized virion building blocks”^4^. We could not discard the hypothesis that these structures might be premature or defective particles, as the occurrence of abnormal particles has previously been reported for other viruses, including giant viruses^26,35^. Notably, the quantification of occurrence of these misshapen structures revealed that 7% of the cells presented at least one of these elements detected in different regions of the host cytoplasm alongside mature virions; thus, these particles are not confined to a viral factory, suggesting that these elements might be defective particles and not particles under morphogenesis.

### Viral progeny present ovoid-shaped format, striped capsid and size plasticity

**Figure 6:**
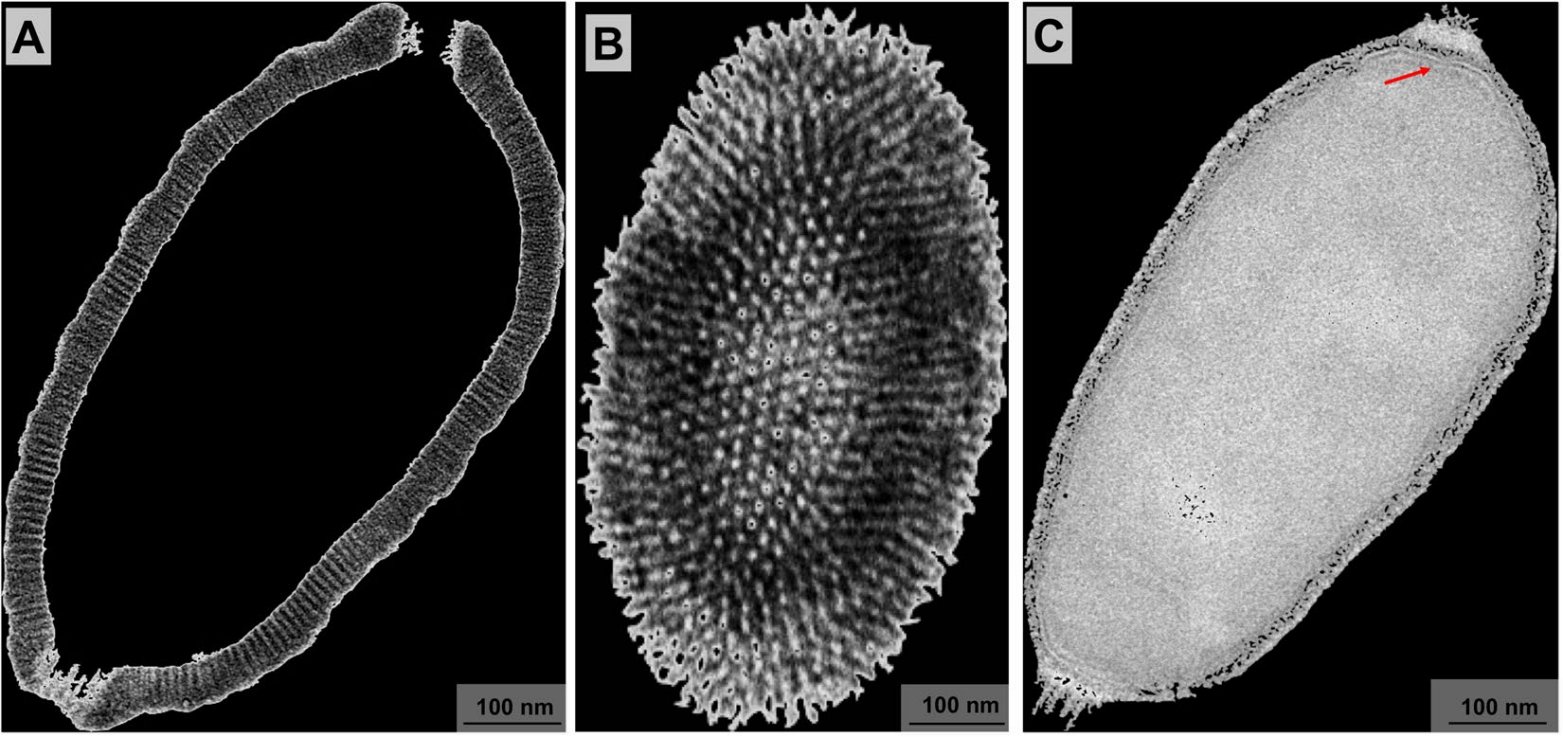
Cedratvirus getuliensis particles present a striped amphora-shaped format and a size plasticity. (**A**) Typical capsid presenting parallel stripes and not completely opposite corks. (**C**) Superficial section of a mature particle evidencing the striped wall. (**D**) Capsid interior composed by a membrane (red arrow) that delimits the internal homogeneous compartment. Images were obtained by TEM and graphically highlighted by using IOS image visualization software.

As mentioned, the end of the C. getuliensis replication cycle is characterized by cell lysis with the consequent release of viral particles. An observation of the viral progeny revealed mature particles measuring ~1 µm in size and ~0.5 µm in diameter and showing an ovoid-shaped format with a typical capsid presenting parallel stripes (Fig. 6A). We sagittally sectioned the lateral top, revealing that the virion subunits appear as organized dots (Fig. 6B). Inside this capsid, we observed a putative membrane delimiting the internal compartment without substructures (Fig. 6C). We believe that this putative inner membrane is acquired during the first steps of morphogenesis, prior to the filling of the particles with the viral genome and virion proteins. Unlike that observed for pithovirus, the interior of Cedratvirus getuliensis virions does not harbor episodic electron-dense spheres or tubular structures but is rather homogeneous^4^.

As a hallmark of cedratvirus virions, C. getuliensis particles also showed two characteristic protruding striped corks at each apex (Fig. 6A and C). However, although these corks are located at the apices, these structures are not antipodally aligned to each other (Fig. 6A and B) and we observed the existence of a misalignment between the centers of the opposite corks.

Although most of the C. getuliensis particles present a similar morphological pattern, different mature particles were also present. This variation is primarily related to the size of the particles, as shown by scanning electron microscopy analyses that revealed the presence of virions up to 2.04 µm, almost the double the size observed for the majority of particles. Therefore, these data provide evidence of size plasticity for the progeny of Cedratvirus getuliensis, as demonstrated for Pithovirus sibericum^36^.

### Cedratvirus getuliensis virions can be released after cell lysis or by exocytosis

**Figure 7:**
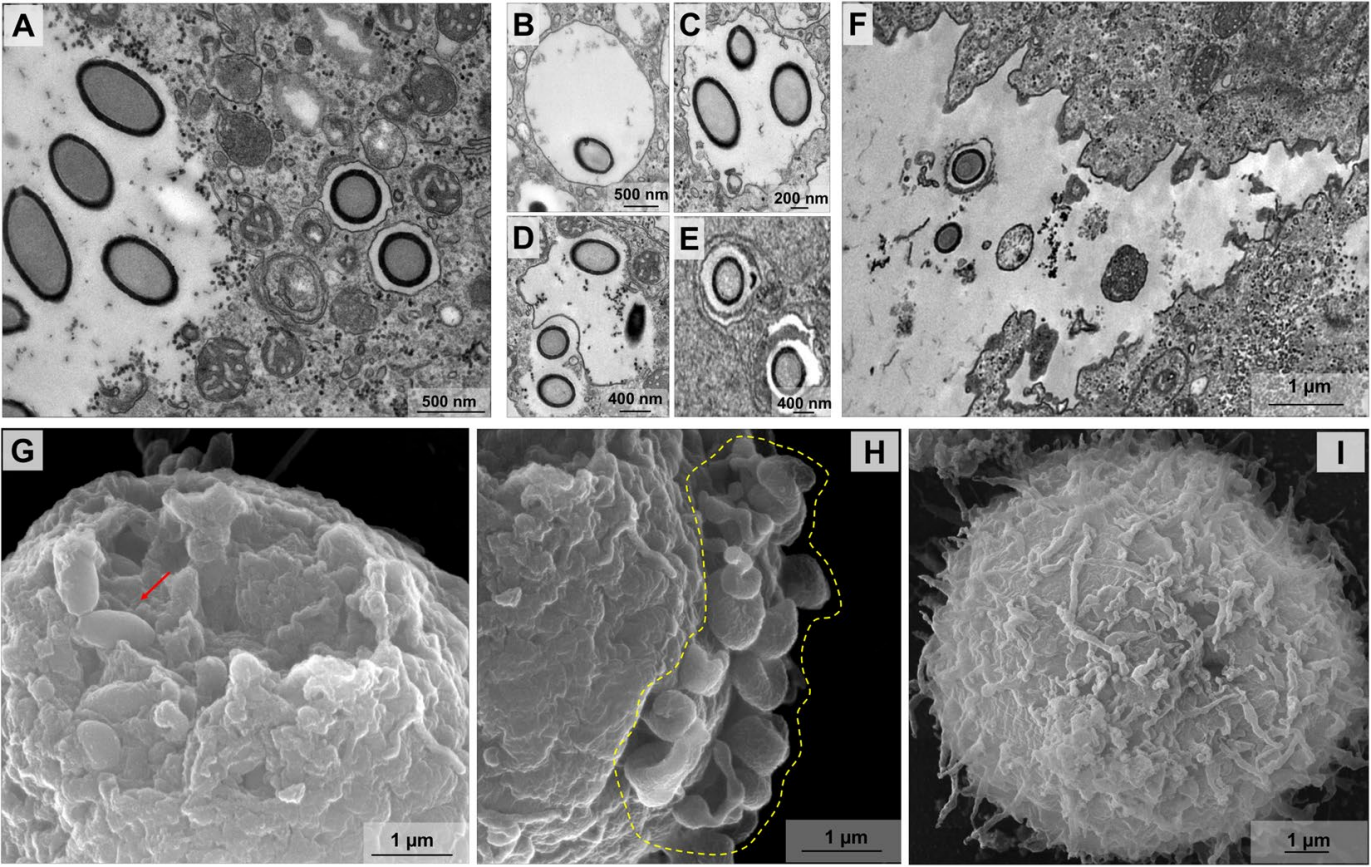
Cedratvirus getuliensis virions can be released after cell lysis or by exocytosis. (**A**) New Cedratvirus getuliensis particles are found immersed in the host cytoplasm or inside vacuoles. (**B–C**) Vacuoles presenting one or more visible particles. (**D**) Particles being engulfed by a membrane. (**E**) Vacuole with more than one membrane. (**F**) Particle insides vacuole apparently outside the cell membrane. (**G**) Scanning microscopy of a host cell presenting a huge damage in the membrane from where new viral particles were released (red arrow). (**H**) Many blebs in the plasma membrane can be observed at the end of infection. (**I**) Cell control not presenting blebs formation in membrane.

After the capsid thickening process, the viral morphogenesis and maturation is now complete and new virions are found immersed in the host cytoplasm surrounded by a halo that, despite could be an artefact of epon embedding, is recurrently observed around other giant viruses particles studies^4,6,10,12,37^. Furthermore, new viruses were also observed embedded within membranes (Fig. 7A). Interestingly, these data revealed the presence of one or more particles inside the same vacuole (Fig. 7B,C and D), which could also present more than one membrane (Fig. 7E). We also observed some particles insides vacuoles and outside the cell membrane, but based only in a 2D perspective we could not affirm that the particles are indeed outside of the amoebas or inside some membrane protrusions (Fig. 7F). The presence of the giant virus progeny inside vacuoles has previously been described for Pithovirus sibericum, suggesting that these particles could be released from the cell by exocytosis^4^. Although exocytosis could be an alternative mechanism used for releasing viral progeny, the main strategy used for Cedratvirus getuliensis is cell lysis. Scanning electron microscopy analyses of the late steps of the C. getuliensis cycle reveals the presence of many cells with substantial damage in the plasmatic membrane, where new viral particles are released (Fig. 7G). Furthermore, the cell lysis is accompanied by plasma membrane blebbing (Fig. 7H), that was not visible in control cells not infected by C. getuliensis (Fig. 7I). However, the causes of these blebs induced upon Cedratvirus getuliensis infection deserve further investigation.

## Discussion

The current understanding of the virosphere has dramatically changed after the discovery of mimivirus, which paved the way for the discovery of other giant and complex amoeba-infecting viruses^29^. Although many studies have highlighted that giant viruses can be phylogenetically related and may form a new putative viral order ‘Megavirales’ along with other large DNA viruses^38^, these viruses present a plethora of virion structures and remarkable differences regarding their developmental cycles. In the present study, we present the first in-depth description of Cedratvirus getuliensis replication cycle, providing valuable information to better understand the biology of this new group of viruses.

Cedratviruses are ~1.4 µm in size and ~0.5 µm in diameter, representing one of the longest viruses described thus far, along with their close relative pithoviruses^4,10,12,34^. Due to their huge size, it was initially proposed that these viruses started their replicative cycle by entering the hosts through phagocytosis, but no experimental data was provided to support this hypothesis, except for a few microscopy images. Here, we demonstrated that the inhibition of phagocytosis with cytochalasin D results in a reduction of Cedratvirus getuliensis virion incorporation by amoebas, suggesting that this virus may enter by this pathway (Fig. 1B,C and D). However, the inhibition of macropinocytosis by EIPA does not affect the entry of Cedratvirus getuliensis particles (Fig. 1B). Interestingly, *Acanthamoeba* cells treatment with chloroquine increased C. getuliensis viral titer, suggesting that this inhibitor could accumulates inside the phagosomes, resulting in pH increasing and consequent prevention of uncoating process; thus preserving a higher number of not uncoated virions inside phagosomes. Following entry, one of the corks is expelled, enabling the fusion of the internal membrane with the phagosome membrane and further releasing the genome into the host cytoplasm^10^. The precise mechanism that triggers these events remains unclear, but it may be related to the low pH environment of phagosomes, similar to the mechanism observed for mimiviruses^26^.

**Figure 8:**
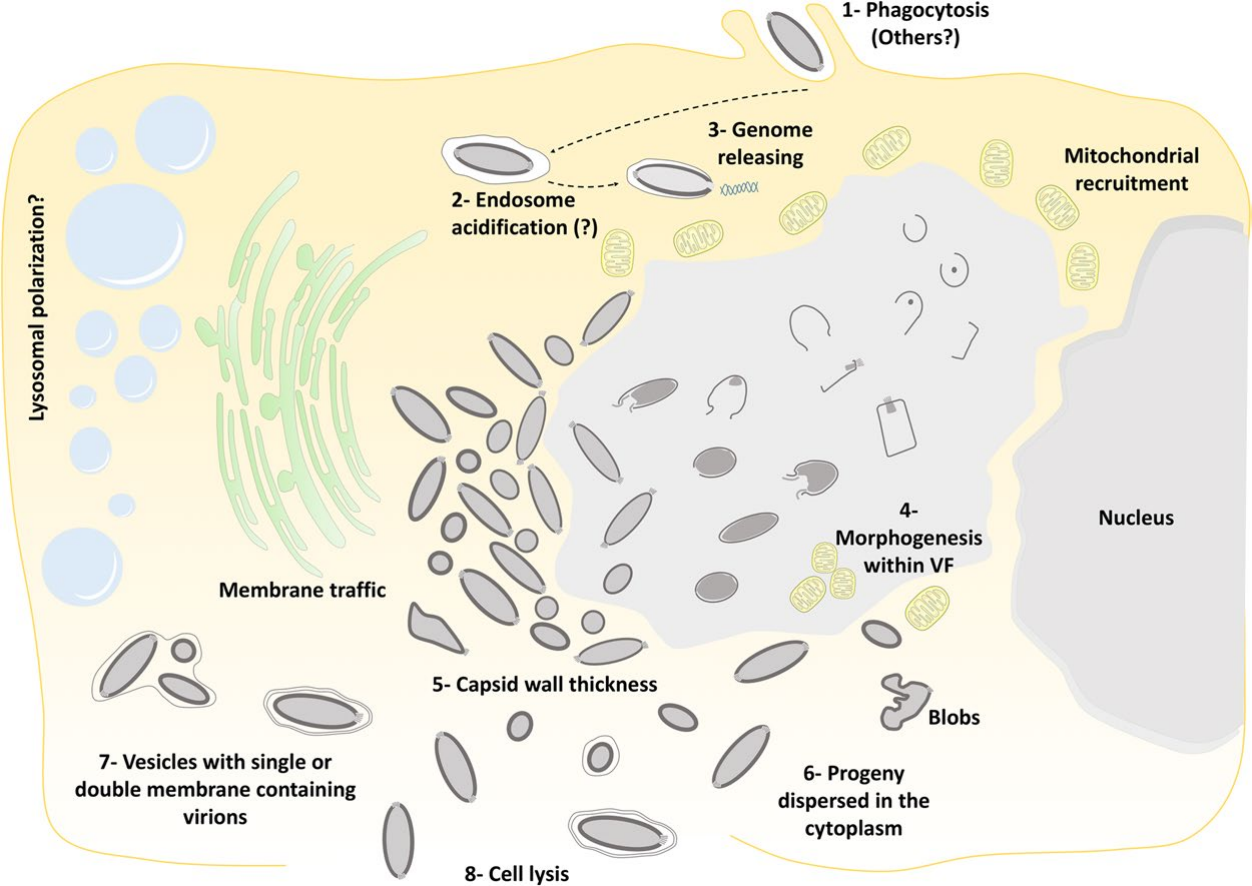
Schematic representation of the Cedratvirus getuliensis replication cycle. Infectious particles enter their host cells by phagocytosis (1). Following entry, one cork is expelled and the fusion of the internal membrane with the phagosome membrane occurs with the release of the genome into the cytoplasm, as demonstrated by Andreani *et al*. (2016) and Bertelli *et al*. (2017) (2–3). The precise mechanism that triggers the cork expelling and membrane fusion remains unknown, but it may be related to the low pH environment of phagosome. After an eclipse phase, a large electron-lucent viral factory is formed, wherein the virion morphogenesis occurs (4). This process is complex and involves sequential structures starting with crescent-shaped precursors, followed by longitudinal-elongation, first cork acquisition, and the presence of structures with a horseshoe-like and rectangular shape (4). Next, a progressive filling of the capsid is observed, followed by the complete closure of the capsid and the emergence of the second cork (4). Thickening of the capsid wall (5) also occurs, and new viral particles are observed dispersed throughout the cytoplasm (6) or taken up by vesicles with one or two membranes (7). The viral progeny are primarily released through cell lysis (8), but exocytosis is also likely to occur.

After an eclipse phase, a large electron-lucent viral factory (VF) is formed, wherein genome replication and virion morphogenesis occur. It is still uncertain whether the host nucleus is involved in the replication of the cedratvirus genome, since the nucleus remains apparently unaltered during the entire viral cycle, different from other giant viruses^6,11^. Similar to pithovirus, no delimiting structure was observed around the VF of Cedratvirus getuliensis, which is perinuclearly located^4^. Cedratviruses present a gene-set related to DNA replication and transcription^10,12^, and it is possible that these elements are packaged into mature virions, similar to its closest relative Pithovirus sibericum^4^; no nuclear machinery is required during cedratvirus replication, in contrast to that described for marseillevirus^39^. The morphogenesis of cedratviruses is complex, wherein different structures are observed until the full maturation of the virion, which exclusively occurs within the VF (Figs. 3 and 4). Similar to other large and giant DNA viruses, cedratviruses form crescent-like structures and may exhibit an internal membrane, although its origin is still unknown^15,26,30–33,39^. Besides to this putative intern membrane, we also observed transversal-sectioned capsids been filled with an electron-dense material that suggest the occurrence of genome and virion protein acquisition (Fig. 3G,H and I). The complete morphogenesis of the virion resembles that of pithoviruses, with a rectangular shape initially emerging, followed by a thickening of the capsid and subsequent acquisition of an oval shape^4^; but differently from its relative, cedratviruses acquire a second cork at the end of the process. Furthermore, no horseshoe structure has been described for pithoviruses. It is likely that this feature is shared by the members of the putative ‘*Pithoviridae*’ family, but additional studies on the morphogenesis of pithoviruses are needed to corroborate this hypothesis. The replication cycle is completed with the release of new viral particles primarily through cell lysis, but exocytosis is likely to occur, since we observed some viral particles embedded in the membranes and outside the host cells. The origin of these membranes is not clear, but we observed that treatment with BFA significantly impacted the viral titer, showing that membrane traffic is important for the occurrence of virion morphogenesis and/or exocytosis. Although no specific labeling for lysosomes was used, we observed the polarization of structures that resemble these organelles during cedratviruses infection, that could suggest the occurrence of autophagy of target viral components or virions, once this organelle acts as an end point degradative structure (Fig. 2)^40^. Moreover we also noted the recruitment of mitochondria, which could be related to the optimization of energy acquisition, required for viral replication (Fig. 2)^40^. However, the actual impact of these organelles on the viral replication cycle remains unknown. Finally, based on the present data, we provide a general view of the entire life cycle of cedratviruses (Fig. 8 – see legend for details).

There are still some unanswered questions concerning the replication cycle of this new group of viruses, especially at the molecular level. Further investigation using different imaging techniques, combined with transcriptomics and proteomics data, will certainly provide valuable insights into the virus-host interaction dynamics and fill some remaining gaps concerning the life cycle of cedratviruses. The world of giant viruses is constantly increasing, and investigating their infectious biology will provide a better understanding of the ecology and evolution of these complex organisms.
